# Application of LAMP and TaqMan qPCR for the rapid diagnosis of *Anaplasma Capra* (an emerging tick-borne zoonotic pathogen) and comparison with Nested-PCR

**DOI:** 10.1007/s11259-026-11074-x

**Published:** 2026-02-09

**Authors:** Kursat Altay, Ufuk Erol, Omer Faruk Sahin, Husnu Furkan Sakar

**Affiliations:** 1 Department of Parasitology, Faculty of Veterinary Medicine, Sivas Cumhuriyet University, Sivas, 58140 Türkiye; 2 Department of Parasitology, Institute of Health Sciences, Sivas Cumhuriyet University, Sivas, Türkiye

**Keywords:** Anaplasma capra, GroEL, Nested-PCR, LAMP, TaqManqPCR, Field samples

## Abstract

*Anaplasma capra* was first detected in goats from China in 2012, and in the process, its presence has been demonstrated in humans, domestic animals, wild animals, and ticks in three continents (Asia, Europe, and Africa). Although there is limited information, it is thought that the agent may cause clinical symptoms in humans and animals. This study aimed to develop two molecular diagnostic assays [Taqman real-time PCR (qPCR) and Loop-Mediated Isothermal Amplification (LAMP)] that can be used in the diagnosis of *A. capra*, including known genotypes, by targeting the *groEL* gene, and to compare the limit of detection (LoD) and the effectiveness in field samples with nested-PCR. The detection limits of *groEL*-nested-PCR, qPCR, and LAMP methods were also investigated. PCR, qPCR, and both colorimetric and conventional LAMP assays were able to identify 8.25 × 10^4^, 8.25 × 10^3^, and 8.25 × 10^2^ copies of *A. capra* DNA/µL in the sample, respectively. According to this finding, qPCR and LAMP had detection limits that were 10 and 100 times greater than PCR’s, respectively. As a result of 150 field samples (sheep, goat, cattle, buffalo, dog, and cat) analyses, two (1.33%) positives in nested-PCR, four (2.66%) positives in qPCR, and five (3.33%) positives in LAMP were detected. The level of agreement between assays was evaluated using Cohen’s kappa test, and consistency was observed between the methods. In conclusion, this study has demonstrated that LAMP and qPCR, which are being used for the first time to diagnose *A.capra* by targeting the *groEL* gene, are more sensitive than nested-PCR. These tests, which have a high level of sensitivity, will be beneficial in understanding the pathogen’s epidemiology.

**Supplementary Information:**

The online version contains supplementary material available at 10.1007/s11259-026-11074-x.

## Introduction

*Anaplasma capra* (genus: *Anaplasma*, family: Anaplasmataceae) is a gram-negative, obligate intracellular bacterium (Li et al. 2015; Peng et al. 2021). This pathogen was first identified in goats in China in 2012 as uncultured *Anaplasma* sp. (Liu et al. 2012). Subsequently, in 2015, the pathogen was identified in people with a history of tick bites in China by Li et al. (2015). The near-full sequence of 1,499 bp 16 S rRNA gene showed 27–73 nucleotide differences compared to other *Anaplasma* species, and the isolate was designated as *A. capra* (Li et al. 2015). In studies conducted by Khumalo et al. (2018) and Shi et al. (2020), it was determined that *A. centrale* identified in cattle and deer and *Anaplasma* sp. detected in serow were *A. capra* based on phylogenetic analyses of the 16 S rRNA gene, highlighting that this species had been circulating undetected for years among hosts. The zoonotic potential of *A. capra* was identified within just three years of its discovery, much faster than the approximately 60 years it took for *A. phagocytophilum*. The pathogen has since been detected in several domestic animals (cattle, sheep, horses, buffalo, dogs, and cats) and wildlife species, including various deer and onagers [reviewed by Altay et al. (2024a)].

*Anaplasma* species are classified as tick-borne pathogens (TBPs) due to their biological transmission through ixodid ticks (Dumler et al. 2001; Dumanli et al. 2012). Nevertheless, they can also be transmitted through contaminated surgical instruments, other blood-sucking arthropods, or even transplacental (Scoles et al. 2005a, b; Kocan et al. 2010; Aubry and Geale 2011). *A. capra* was initially identified in *Ixodes persulcatus* by Li et al. (2015). Since then, it has also been detected and reported in both engorged and host-seeking ticks, such as *Haemaphysalis longicornis* (Yan et al. 2021), *Hae. qinghaiensis* (Han et al. 2019), *Rhipicephalus microplus* (Guo et al. 2018), I. *kashmiricus* (Numan et al. 2023), *Dermacentor everestianus* and *D. nuttalli* (Han et al. 2019). Additionally, *A. capra* was detected in the salivary glands of a female *Hae. concinna* tick (Jouglin et al. 2025). Although current studies have documented the presence of *A. capra* in ticks, their vector competence should be confirmed with experimental studies.

The clinical manifestations of *A. capra* infection in its hosts remain poorly characterized due to the limited number of reported cases. Shi et al. (2019) reported a direct association between *A. capra* infection and anemia in dogs, while Staji et al. (2021) observed mild reductions in red blood cell, hematocrit, and hemoglobin levels, along with leukopenia, lymphopenia, thrombocytopenia, hypoalbuminemia, and hyperbilirubinemia in two Persian onagers. To date, only one study has described *A. capra* infection in humans, with clinical manifestations, including influenza-like symptoms (fever, malaise, headache, dizziness, and chills), gastrointestinal symptoms (nausea, vomiting, or diarrhea), rash, eschar, and regional lymphadenopathy. These cases were managed with doxycycline therapy (Li et al. 2015).

After it was understood that *A. capra* threatened human and animal health, numerous epidemiological studies were conducted, the pathogen has been reported in approximately 20 countries, including Angola, China, Ghana, Greece, France, India, Iran, Japan, Kyrgyzstan, Malaysia, Morocco, Pakistan, South Korea, Spain, Sweden, and Türkiye (Kawahara et al. 2006; Liu et al. 2012; Seo et al. 2018; Koh et al. 2018; Grandi et al. 2018; Jouglin et al. 2019; Staji et al. 2021; Barradas et al. 2021; Elhachimi et al. 2021; Altay et al. 2022a, b, c; Ishaq et al. 2022; Remesar et al. 2022; Saratsis et al. 2022; Addo et al. 2023; Kumar et al. 2023), suggesting that *A. capra* is a cosmopolitan species. Accurate epidemiological data are essential for effective disease control. To achieve these data, it is necessary to employ identification methods with high specificity and sensitivity to investigate the pathogens in intermediate and definitive hosts. However, studies conducted to date have shown that only conventional PCR methods have been used in the diagnosis of *A. capra*, and that different molecular-based methods have not been used (Koh et al. 2018; Jouglin et al. 2019; Staji et al. 2021; Barradas et al. 2021; Elhachimi et al. 2021; Altay et al. 2022a; Ishaq et al. 2022; Remesar et al. 2022; Addo et al. 2023; Kumar et al. 2023; Lin et al. 2023; Sahin et al. 2023). Molecular-based methods (such as LAMP and qPCR), which have higher specificity and sensitivity compared to the conventional PCR method (Notomi et al. 2000; Kubista et al. 2006; Bustin et al. 2009), can be used for the diagnosis of *A. capra*. Aims of this study were; (i) to apply LAMP and TaqMan qPCR methods targeting the *groEL* gene, which this gene is more informative than the 16 S rRNA gene, both in distinguishing *Anaplasma* species and in determining intraspecific genetic diversity of *A. capra* (Lew et al. 2003; Li et al. 2015; Yang et al. 2016a; Khumalo et al. 2018; Sahin et al. 2022), (ii) to determine specificity, sensitivity, limit of detection of LAMP and TaqMan qPCR assays, (iii) to assess diagnostic performance of these assays in field samples obtained from various hosts species.

## Materials and methods

### *Anaplasma capra groEL*-LAMP assays

#### Design of *Anaplasma capra groEL*-specific LAMP primers

Before designing LAMP primers, all *A. capra groEL* gene sequences available in GenBank were analyzed. A total of 205 *groEL* sequences isolated from cattle, sheep, goat, buffalo, dog, cat, human, red deer, swamp deer, roe deer, Persian onager, Korean water deer, *R. microplus*,* Hae. longicornis*,* Hae. qinghaiensis*, and *D. everestianus* were retrieved from GenBank; however, 192 were suitable for primer design, because the remaining sequences were too short to cover the target region. The sequences were aligned using the MEGA-11 software (Tamura et al. 2021). The nucleotide sequence of *A. capra* (GenBank accession number ON783820), selected as a representative of the 192 aligned sequences, were used for primer design. The primer sets were designed (Table 1) using the online program (NEB LAMP primer design tool (https://lamp.neb.com/#!/). The primers’ specificities were analyzed using the BLASTn algorithm.

**Table 1:** *A. capra groEL-*specific LAMP primers

The name of primers	Nucleotide sequences (5’−3’)
AcpF3	TCGCAATGCAACGATAAGGT
AcpB3	GCGGATATRGTGGCAACCTG
AcpFIP	GTATCAGCGCCAGCAGCCTTG - CRTGYTCCATACTCACCGCA
AcpBIP	TGGGATTCTGAARGCYAAGGARG - CTTCAGAYRCAACTTCRCGC
AcpLoop F	CYTTRGCMACTTCCTCTATRACCTT
AcpLoop B	GCCGCCYTRCTGTCAATGAA

### Optimization of *Anaplasma capra groEL*-LAMP (Conventional and Colorimetric LAMPs)

Optimal incubation temperature is critical for LAMP efficacy. The LAMP mix was incubated at several temperatures (58 °C to 65 °C) for 60 min to determine the optimal amplification temperature. *A. capra* positive DNA that was obtained from a water buffalo (*Bubalus bubalis*) blood sample (GenBank accession number: ON783820) and negative (DNase-RNase-free sterile water, Cat No.: 129114, Qiagen, Germany) controls were employed during the optimization process of both conventional and colorimetric LAMP assays.

The conventional *groEL*-LAMP was performed using Bst II Pro DNA Polymerase Large Fragment kit (Cat. No: P703-02, Vazyme). Conventional LAMP master mix was prepared in a final 25 µL reaction volume using; 2.5 µL 10× IsothermalAmp Buffer, 1.5 µL MgSO_4_ (100 mM), 3.5 µL dNTP Mix (10 mM each) (Cat. No: R0192, Thermo Scientific, Lithuania), 1.6 µM FIP, 1.6µM BIP, 0.2 µM F3, 0.2 µM B3, 0.8 µM LoopF, 0.8 µM LoopB, 0.32 U/µL Bst II Pro DNA Polymerase Large Fragment (8 U/µL), 1 µL template DNA (GenBank accession number: ON783820). 14.2 µL DNase/RNase-free distilled water (Cat No: 129114, Qiagen, Germany) was added to make the final reaction volume 25 µL. The amplification was performed in a PCR thermal cycler (TurboCycler (TCST-9622), Blue Ray Biotech, Taiwan) at 58–65 °C for 60 min, and a 10-minute incubation at 90 °C was applied for the enzyme inactivation. The LAMP amplification products were visualized on a 2% agarose gel stained with ethidium bromide after gel electrophoresis. The best amplification temperatures were recorded.

The colorimetric *groEL*-LAMP was carried out using WarmStart Colorimetric LAMP 2× Master Mix (Cat. No: M1800, New England Biolabs). Colorimetric LAMP master mix was done with a volume of 25 µL using; 12.5 µL WarmStart Colorimetric LAMP 2× Master Mix, 1.6 µM FIP, 1.6 µM BIP, 0.2 µM F3, 0.2 µM B3, 0.8 µM LoopF, 0.8 µM LoopB, 1.6 µM FIP, 1.6 µM BIP, 0.2 µM F3, 0.2 µM B3, 0.4 µM LoopF, 0.4 µM LoopB, 1 µL template DNA (GenBank accession number: ON783820). 9 µL DNase/RNase-free distilled water (Cat No: 129114, Qiagen, Germany) was added to make the final reaction volume 25 µL. The amplification was performed in the same temperature and time conditions as the conventional *groEL*-LAMP described above. The results of the colorimetric LAMP assay were evaluated visually according to color shift (pink to yellow in positive samples), and recorded.

### *Anaplasma capra groEL*-TaqMan qPCR

#### Design of *Anaplasma capra groEL*-specific qPCR primers and prob

TaqMan qPCR primers and one probe were designed to target an 89 bp fragment of the *groEL* gene of *A. capra* (Table 2). During the design of primers and probe, the same sequences described in the section “Design of *Anaplasma capra groEL*-specific LAMP primers” were used. Following sequence analysis in MEGA-11 (Tamura et al. 2021), primers and probe were designed using the Real-time PCR (TaqMan) Primer and Probes Design Tool (https://www.genscript.com/) and the PrimerQuest Tool (https://eu.idtdna.com/). Primers and probes were evaluated using OligoAnalyzer (https://eu.idtdna.com/calc/analyzer) in accordance with their GC content, hairpin formation, and the melting temperature values. The most appropriate sequences were identified. The specificity of the primers and probe were evaluated using the BLASTn algorithm.

**Table 2:** A. capra groEL-specific TaqMan qPCR primers and probe

The name of primers and probe	Nucleotide sequence (5’−3’)
Acp TaqMan-F	GCAAAGGGTTCAAAGACCTMGA
Acp TaqMan- Probe	FAM-TGCAGTTYGATCGCGGATACCTTT-BHQ
AcpTaqMan-R	CTCAAACTCTACCAGCATCTTCTC

### Optimization of *Anaplasma capra groEL*- TaqMan qPCR

The *A. capra groEL*-specific TaqMan qPCR assay was performed using the TaqMan probe-based qPCR kit (BlasTaq™ Probe 2× qPCR MasterMix, abm^®^, Canada). The reaction mixture was prepared according to the manufacturer’s instructions, and the method was run at varying primers (200nM-500nM) and probe (100nM-300nM) concentrations to determine their optimal concentrations. *A. capra* positive DNA (GenBank accession number: ON783820) was used in the assay. The TaqMan qPCR assay was run after enzyme activation at 95 °C for 3 min, followed by 40 cycles of denaturation at 95 °C for 15 s and at 58 °C for 1 min. Amplification curves were controlled, and the required primer concentration was picked to create an efficient amplification curve. Furthermore, the TaqMan qPCR method was also carried out to determine the optimal annealing temperature. For this purpose, TaqMan qPCR assay was run at 95 °C for 3 min, followed by 40 cycles of denaturation at 95 °C for 15 s and annealing at varying temperatures (57–60 °C) for 1 min. The reaction mixture was prepared according to the manufacturer’s instructions (BlasTaq™ Probe 2× qPCR MasterMix, abm^®^, Canada), 300 nM primer and 150 nM probe were used in the final volume of the qPCR mixture. Amplifications was done using the Q1000 + Real-Time System (LongGene, China). Samples with a quantification cycle (Cq) value ≤ 35 were considered positive. *A. capra* positive DNA that was obtained from water buffalo blood (*Bubalus bubalis*) sample (GenBank accession number: ON783820) and negative (DNase-RNase free water, Cat No: 129114, Qiagen, Germany) controls were included in the above-mentioned TaqMan qPCR assays.

### Determination of specificities of LAMP and TaqMan qPCR assays

To evaluate assay specificity, positive control DNAs from common tick-borne haemoparasite species were included. The corresponding details are listed in Table 3.

Prior to conducting the specificity assays, the positive control DNA samples listed in Table 3 were subjected to PCR with primers AnaplsppF (5′-AGAAGAAGTCCCGGCAAACT-3′) and AnaplR3 (5′-GAGACGACTTTTACGGATTAGCTC-3′) which amplify approximately 800 bp fragment of the 16 S rRNA gene region of species in the *Anaplasma* species (Zobba et al. 2014), and BJ1 (5′-GTCTTGTAATTGGAATGATGG-3′) and BN2 (5′-TAGTTTATGGTTAGGACTACG-3′) which amplify approximately 500 bp fragment of the 18 S rRNA gene region of species in the *Theileria* and *Babesia* species (Casati et al. 2006), to assess their integrity and quality prior to their inclusion in the specificity assays. PCR assays were performed as described by Zobba et al. (2014) and Casati et al. (2006). After the PCR amplification, specific amplicons were visualized by a UV transilluminator following agarose gel electrophoresis. The results were recorded, and the samples were used to determine the specificities of the LAMP (conventional and colorimetric) and TaqMan qPCR assays. LAMPs and TaqMan qPCR assays were performed as described in the relevant sections above.

**Table 3:** Positive control DNA samples used in the specificity analyses of the LAMP and TaqMan qPCR

Species	The type of animal from which it was obtained	The detection method	References
*A. capra*	Cattle	PCR	Altay et al. 2022a
*A. ovis*	Sheep	PCR	Altay et al. 2022b
*A. bovis*	Cattle	PCR	Altay et al. 2024b
*A. marginale*	Cattle	PCR	Altay et al. 2020
*A. centrale*	Cattle	PCR	Altay et al. 2024c
*A. phagocytophilum* like-1 and like-2 mix	Sheep	PCR	Erol et al. 2022
*B. bigemina*	Cattle	RLB	Altay et al. 2020
*B. major*	Cattle	RLB	Altay et al. 2022b
*B. occultans*	Cattle	RLB	Altay et al. 2022b
*T. annulata*	Cattle	RLB	Altay et al. 2022b
*T. orientalis*	Cattle	RLB	Altay et al. 2022b
*Theileria* sp. MK.	Sheep	RLB	Altay et al. 2017
*T. ovis*	Sheep	RLB	Altay et al. 2017
*Theileria* sp. OT3	Sheep	RLB	Altay et al. 2017
*T. equi*	Horse	PCR	Altay et al. 2024c

*PCR* polymerase chain reaction, *RLB* reverse line blotting

### Determination of limit of detection (LoD) of *groEL*-nested PCR, LAMP, and TaqMan qPCR

Recombinant plasmids were constructed by inserting an 874 bp fragment of the *A. capra groEL* gene. Before this process, *A. capra groEL* gene was amplified by nested PCR using Outer f (5′-GCGAGGCGTTAGACAAGTCCATT-3′)/Outer r (5′-TCCAGAGATGCAAGCGTGTATAG-3′) and Inner f (5′-TGAAGAGCATCAAACCCGAAG-3′)/Inner r (5′-CTGCTCGTGATGCTATCGG-3′) (Yang et al. 2016a) to obtain target *A. capra* DNA. Nested-PCR assay was performed as described by Yang et al. (2016a). Specific amplicons were analysed using a UV transilluminator, subsequent to agarose gel electrophoresis following the PCR test.

The amplicon was excised from the agarose gel and purified using a PCR Clean-up & Gel Extraction Kit (Cat. No: NA006–0300, GeneDireX, Taiwan) following the company’s protocol prior to cloning into the plasmid. The purified PCR products were cloned into pJET1.2/blunt cloning vector using CloneJET PCR Cloning Kit (Ref No: K1232, Thermo Scientific, Lithuania) following the Blunt-End Cloning Protocol. Confirmation of the recombinant clones was carried out using PCR amplification of the inserted fragment. The recombinant plasmids were purified using GeneJet Plasmid Miniprep Kit (Ref No: K0502, Thermo Scientific, Lithuania), and stored at −20 °C until the primer detection limits were determined.

To determine the limit of detection of the nested PCR, LAMP, and TaqMan qPCR, the purified recombinant plasmids were quantified using a NanoDrop (Denovix Ds-11, USA). The number of DNA copies in the each sample was calculated using an online programme (https://www.technologynetworks.com/tn/tools/copynumbercalculator) with the following formula:

$$Number\;of\;copies=\frac{DNA\;concentration\;\left(ng/\mu L\right)\times\left[6.022\times10^{23}\right]}{Length\;of\;template\;\left(bp\right)\;\times\;\left[1\times10^9\right]\times650}$$

Samples with a known DNA concentration were serially diluted tenfold to obtain a range of DNA amounts (from 10^0^ to 10^− 10^), which were then used as templates to determine the limits of detection of the methods. Moreover, these samples were also used for determination of the standard curve and TaqMan qPCR efficiency. The efficiency was calculated using the following formula: Efficiency (%) = 100 × (10^− 1/slope^−1). The linearity of data (R^2^ > 0.98), the E value in the 90–110% interval, and the stability of Cq values between five repetitions were considered indicators that the assay was well optimized. In this work, repeatability (intra-assay) was determined from the Cq-values of triplicates using the serially diluted *A. capra* DNA samples. Reproducibility (inter-assay) was determined from the mean Cq-values of five independent experiments using above mentioned samples. Both are referred to as coefficients of variation (CV = SD/mean×100) and were calculated for each concentration.

### Assessment and comparison of the diagnostic performance of nested PCR, LAMP, and TaqMan qPCR methods using field samples

To demonstrate the usability and performace of the *groEL*-LAMP and *groEL*-qPCR assays in field samples and to compare the *groEL*-nested PCR, a total of 150 samples [(25 samples from each animal species: cattle (*Bos taurus*), buffalo (*Bubalus bubalis*), sheep (*Ovis aries*), goats (*Capra hircus*), cats (*Felis catus*), and dogs (*Canis lupus familiaris*)] collected from different regions of Türkiye were tested using with all three methods. In *groEL*-nested PCR, outer and inner primers were used as described by Yang et al. (2016a). For the LAMP and TaqMan qPCR, the samples were processed according to the respective protocols detailed in earlier sections. *A. capra* positive DNA (GenBank accession number: ON783820) and negative (DNase-RNase free water, Cat No: 129114, Qiagen, Germany) controls were included in the nested-PCR, LAMP, and TaqMan qPCR assays to monitor for inhibition and prevent false-positive results.

Levels of agreement between nested-PCR, TaqMan qPCR, and LAMP assays were calculated with Cohen’s kappa test (Cohen 1960), using the following formula;

$$K=\frac{Pr\left(a\right)-Pr\left(e\right)}{1-Pr\left(e\right)}$$

After the calculation, the obtained results were interpreted according to the standard recommended by Landis and Koch (1977).

## Results

### Optimization of *Anaplasma capra* specific diagnostic methods

#### Conventional and colorimetric *groEL*-LAMP

**Fig. 1:**
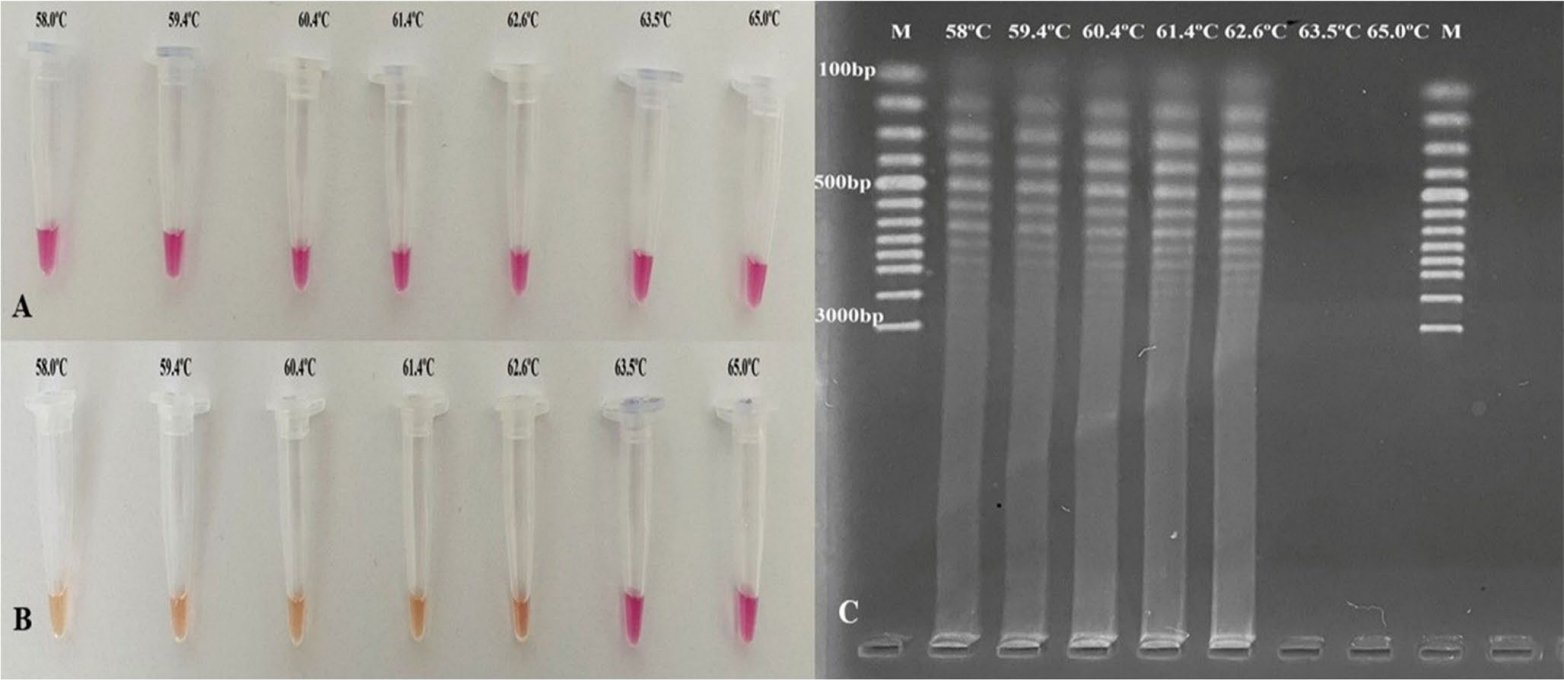
Colorimetric (**A** and **B**) and conventional LAMP (**C**) assays at different temperatures. **A.** The colorimetric LAMP method before being run at different temperatures (58–65 °C) **B**. A color shift from pink (negative) to bright yellow (positive) was observed. **C.** Ladder-like amplicons were seen in samples at appropriate temperatures (58–62.6 °C), and no amplicons were seen in other samples

The reaction temperature and duration were determined for the *A. capra groEL-*colorimetric and conventional LAMP assays. To achieve this objective, temperatures ranging from 58 to 65 °C were implemented. No amplicon was obtained above 62.6 °C (Fig. 1). When the reactions were incubated starting from 30 min and increasing by 10 min, the best results were obtained in 60 min. False positive results began to appear after 90 min of incubation (data not shown). Therefore, all the subsequent analyses were conducted at 62 °C for 60 min.

### *GroEL*-TaqMan qPCR

**Fig. 2:**
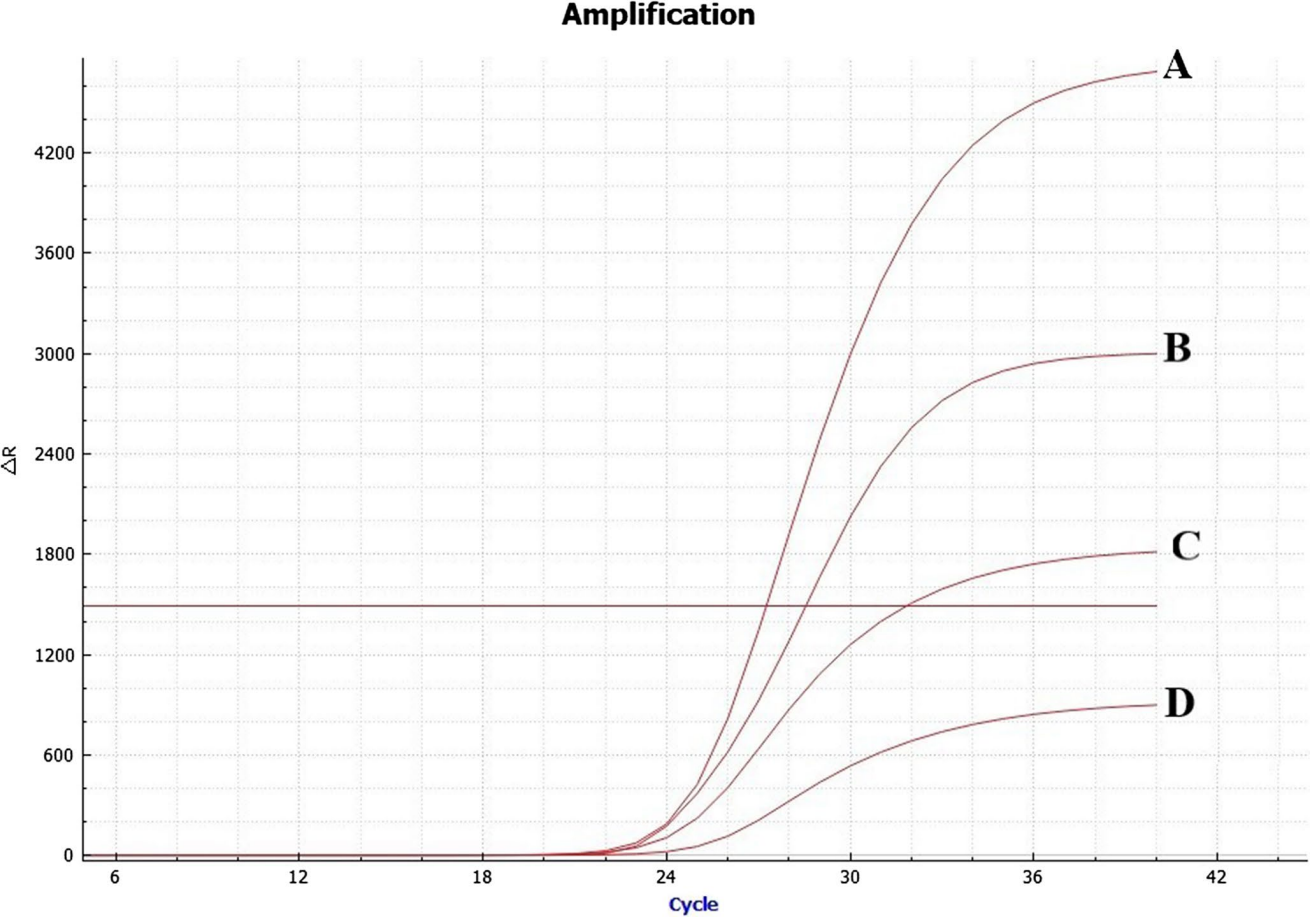
Optimization process of primer and probe concentration for the TaqMan qPCR assay. Curve **A**: 500 nM primers and 300 nM probe concentration, Curve **B**: 300 nM primers and 150 nM probe concentration, Curve **C**: 250 nM primers and 125 nM probe concentration, Curve **D**: 200 nM primers and 100 nM probe concentration

As a result of the optimization performed to determine primer and probe concentrations, it was observed that the positive sample did not pass the threshold value at 200 nM primers and 100 nM probe concentrations. It was seen that positive sample exceeded the threshold value at 31.8 Cq at 250 nM primers and 125 nM probe concentrations. With 300 nM primers and 150 nM probe concentrations, the positive sample passed threshold value at 28.3, while at 500 nM primers and 300 nM probe concentrations, the positive sample exceeded the threshold value at 27.6 (Fig. 2). Considering that there is no significant difference between the concentrations of the last two primers and probes, and that false positives may occur when using high concentrations of primers and probes, it has been decided to use 300 nM primer and 150 nM probe concentrations.

**Fig. 3:**
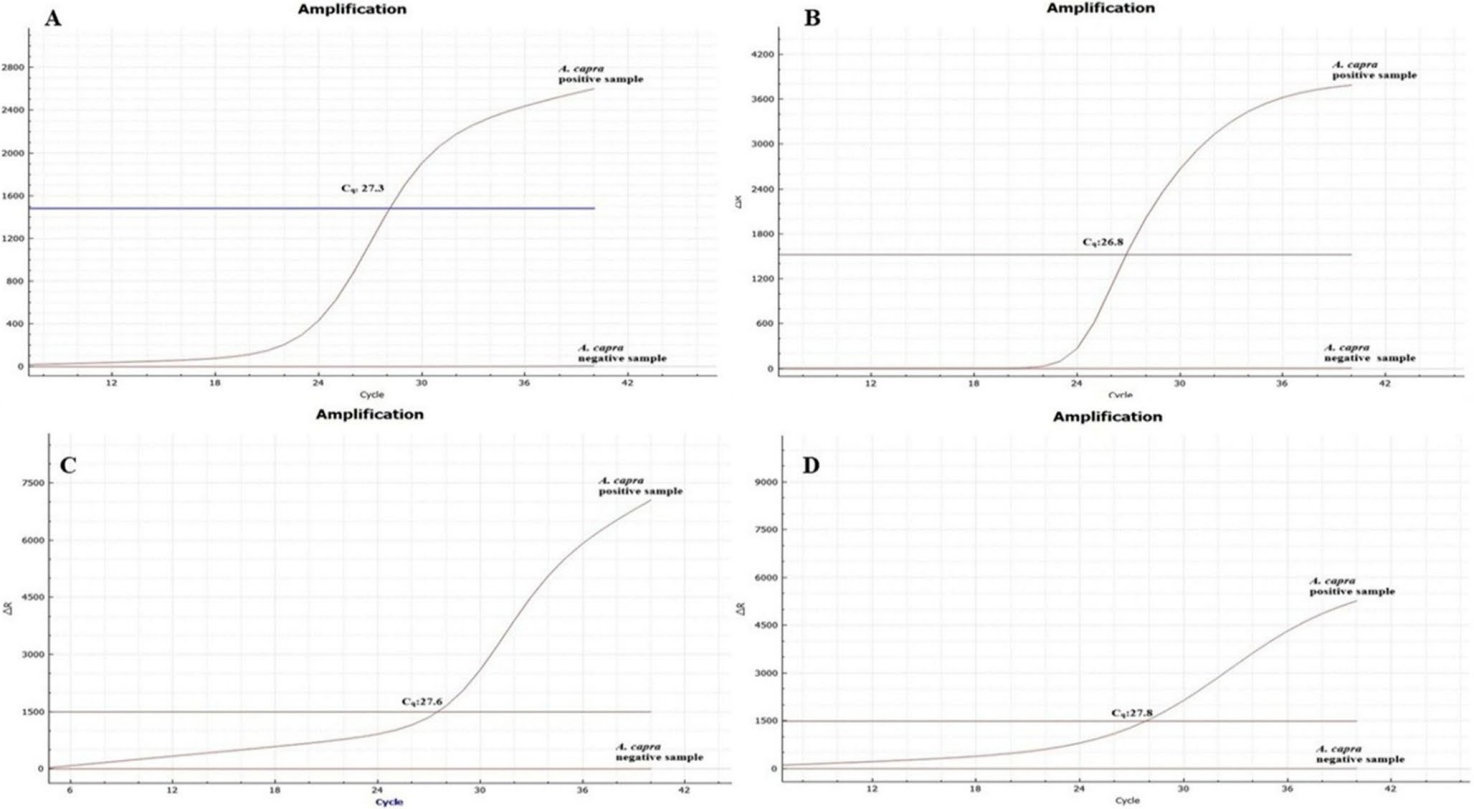
The optimization of annealing temperatures for the TaqMan qPCR using *A. capra* positive and negative samples at different temperatures. **A**: Annealing temperature: 57 °C, **B**: Annealing temperature: 58 °C, **C**: Annealing temperature: 59 °C, **D**: Annealing temperature: 60 °C. A sigmoidal wave was observed in the positive sample

TaqMan qPCR annealing temperature was also optimized, and the optimal temperature was established at 58 °C. It was observed that the positive sample passed the threshold value at 26.8 C_q_, and there was no fluctuation in the negative control sample (Fig. 3). In other temperatures, it was observed that the positive sample exceeded the threshold value at higher C_q_ values (Cq value of 27.3 at 57 °C, 27.6 C_q_ at 59 °C, and 27.8 C_q_ at 60 °C). Consequently, the annealing temperature for the qPCR assay was determined to be 58 °C in accordance with the optimization assay.

### Specificity of conventional and colorimetric *groEL*-LAMP and *groEL*-qPCR assays

The primers and probe developed for the LAMP and TaqMan qPCR (Tables 1 and 2) were initially evaluated using BLASTn. It was revealed that these primers and probe were exclusively specific to the *groEL* gene of *A. capra.*

**Fig. 4:**
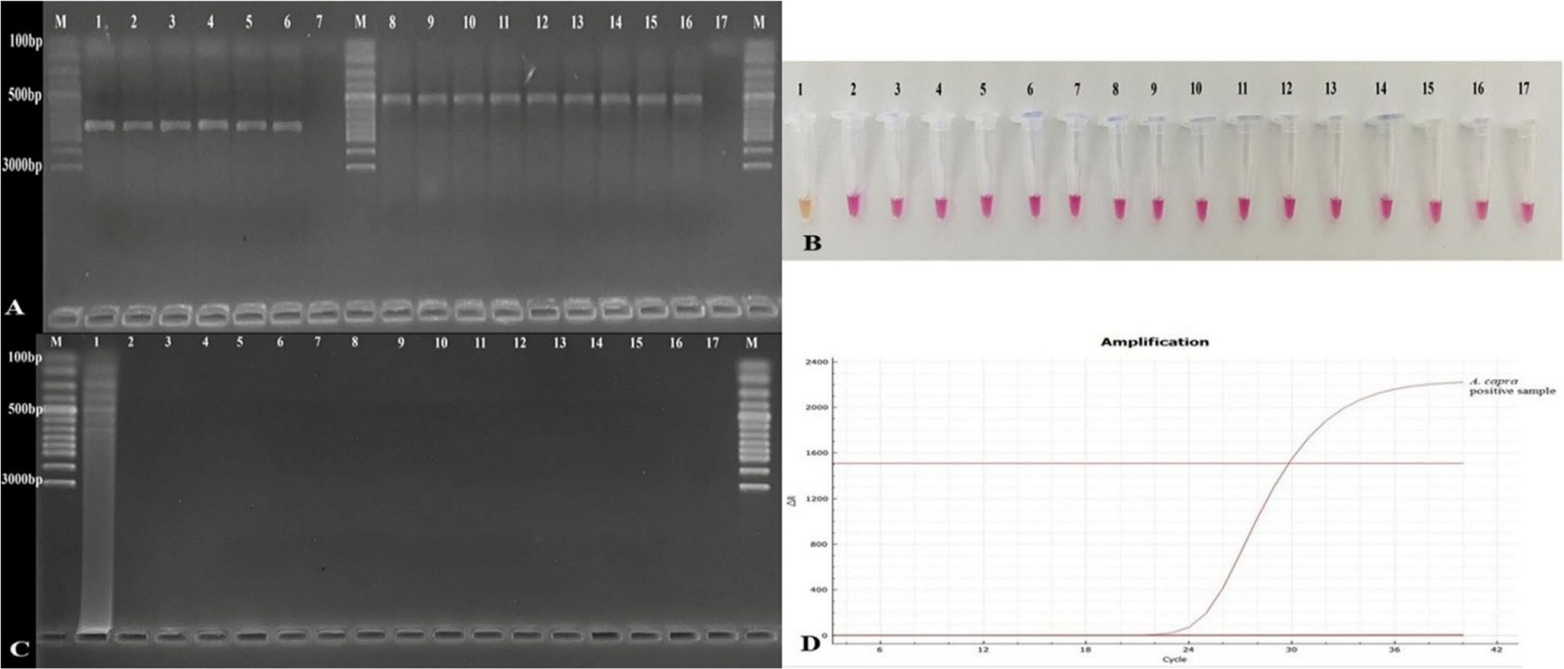
Specificity assays of molecular methods. **A**: (1) *A. capra*, (2) *A. ovis*, 3. *A. bovis*, 4. *A. marginale*, 5. *A. centrale*, 6. *(A) phagocytophilum* like-1 and like-2 mix, 7. *Anaplasma* negative, 8. *(B) bigemina*, 9. *B. major*, 10. *B. occultans*, 11. *T. annulata*, 12. *T. orientalis*, 13. *Theileria* sp. MK., 14. *T. ovis*, 15. *Theileria* sp. OT3, 16. *T. equi*, 17. *Theileria/Babesia* negative. **B**: The colorimetric LAMP gave positive results (color change) for *A. capra* (1), but negative results for other *Anaplasma/Theileria/Babesia* samples. **C**: Conventional LAMP gave positive results (ladder-like amplicons) for *A. capra* (1), but negative results for other *Anaplasma/Theileria/Babesia* samples. **D**: The TaqMan qPCR gave positive results (sigmoidal wave) for *A. capra* (1) and negative results for other *Anaplasma/Theileria/Babesia* species

Furthermore, these primers and probe were also tested against 15 common TBPs (Table 3) isolated from cattle, sheep, and horses, in the laboratory. The DNA samples were checked for DNA degradation prior to use by PCR, and no degradation was detected (Fig. 3A). These 15 DNA samples, known to be positive for different pathogens, were examined using LAMP and TaqMan qPCR. Each method gave positive results exclusively for *A. capra* (Fig. 4B, C, D). These findings indicated that LAMP (conventional and colorimetric) and qPCR assays can be effectively employed to identify *A. capra* in hosts.

### Limit of detection (LoD) of PCR, LAMP, and TaqMan qPCR

**Fig. 5:**
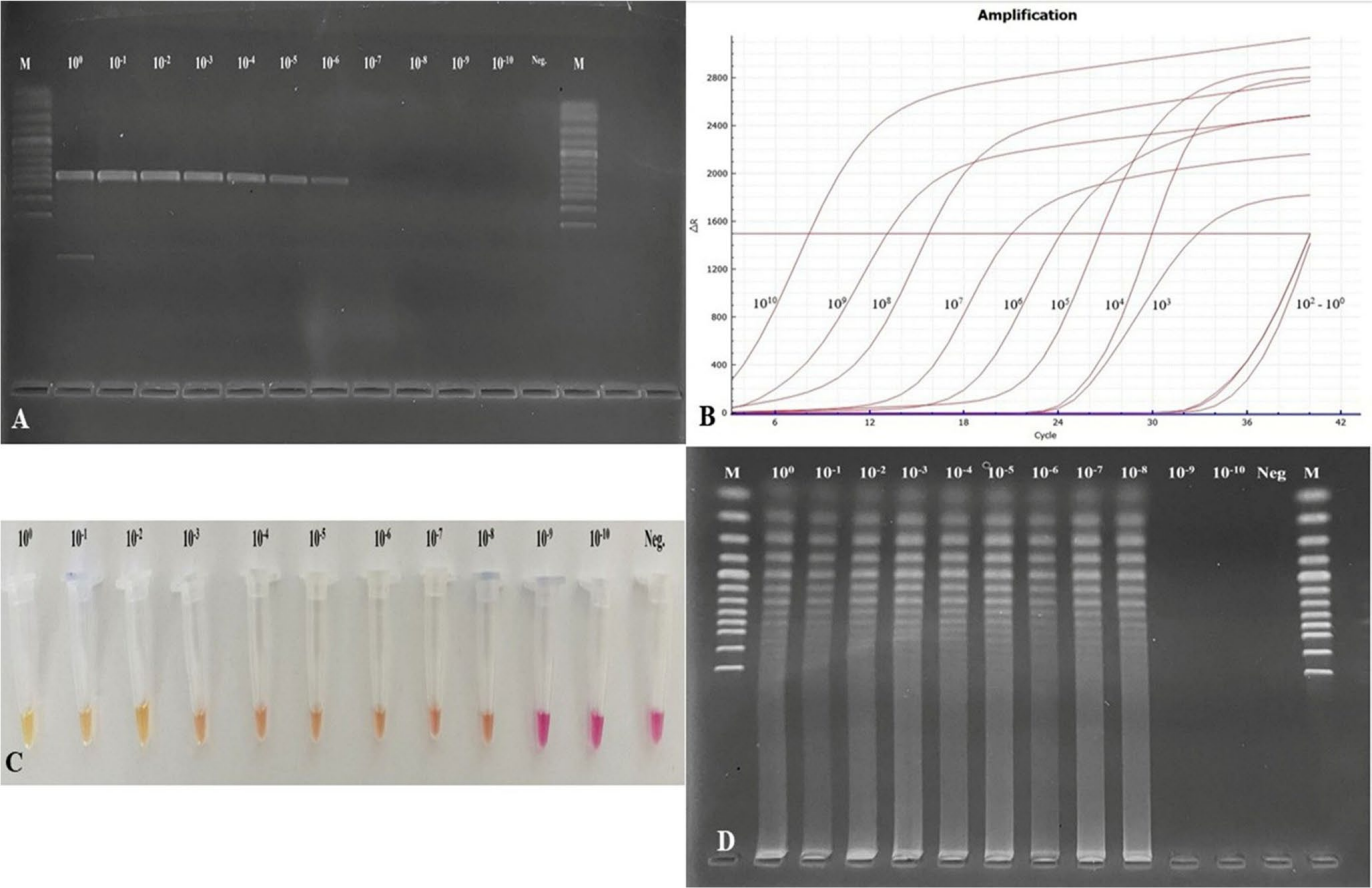
Detection limit of molecular methods. **A**: *groEL*-nested PCR (positive amplicon was up to dilution of 10^− 6^), **B**: *groEL*-TaqMan qPCR (sigmoidal waves were present up to dilution of 10^− 7^), **C**: *groEL*-colorimetric LAMP (color shift was observed up to a dilution of 10^− 8^), **D**: *groEL*-conventional LAMP (ladder-like amplicons were seen up to a dilution of 10^− 8^)

A sample containing 342.66 ng/µL (8.25 × 10^10^ copies of DNA/µL) of *A. capra* DNA was used to determine the limit of detection of the assays. Tenfold serial dilutions (from 10^0^ to 10^− 10^) were performed on the samples, and samples containing different amounts of DNA were obtained. As a result of conventional-PCR assay, positive results were obtained up to 10^− 6^ dilutions (8.25 × 10^4^ copies of DNA/µL) (Fig. 5A). A positive result was detected at 10^− 7^ dilutions (8.25 × 10^3^ copies of DNA/µL) in TaqMan qPCR (Fig. 5B). Color shift was seen up to 10^− 8^ dilutions in the colorimetric method (Fig. 5C), and similarly, the amplicon product was determined in the gel at 10^− 8^ (8.25 × 10^2^ copies of DNA/µL) dilutions in conventional LAMP (Fig. 5D). Consequently, the TaqMan qPCR assay was 10 times more sensitive than conventional PCR. The LAMP assay demonstrated even greater sensitivity, being 100 times more sensitive than PCR and 10 times more sensitive than TaqMan qPCR.

### Efficiency, repeatability and reproducibility of the TaqMan qPCR

**Fig. 6:**
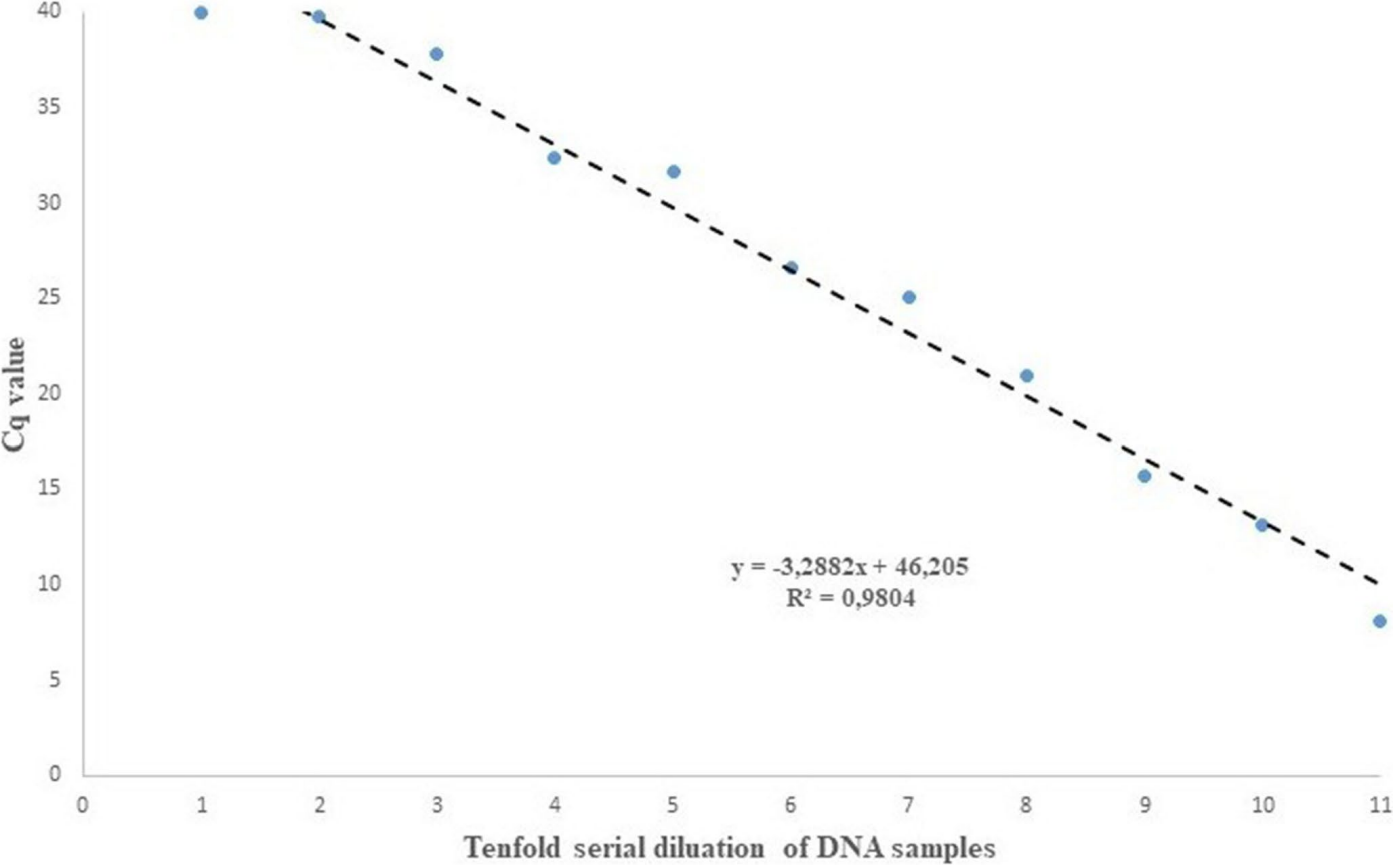
Standard curve demonstrating the linearity of the *A. capra* qPCR test. The efficiency value was calculated as 101.42%

In this work, the linear regression of the method was assessed as R^2^ > 0.98, and the efficiency value was determined as 101.42% (slope of curve:−3.2882) (Fig. 6).

The repeatability and reproducibility of the TaqMan qPCR were evaluated with the inter-run and intra-run standard deviation (SD) and coefficient of variation (CV). The inter-run SD varied between 0.000 and 0.678, and the intra-run SD varied between 0.000 and 0.695. The maximum coefficient of variation (CV%) was between 0.00 and 3.43, suggesting low variance among different repetitions and runs (Table 4).

**Table 4:** Reproducibility (inter-assay) and repeatability (intra-assay) variation values of the TaqMan-qPCR assay for tenfold serial dilutions of *A. capra*. The samples were researched three times in five independent assays (*n* = 15)

Log10 copy number	Mean Cq	Inter-run SD	Intra-run SD	Total SD	Total CV (%)
10^10^	8.149	0.0098	0.068	0.069	0.85
10^9^	13.033	0.419	0.154	0.446	3.43
10^8^	15.420	0.404	0.133	0.420	2.72
10^7^	21.100	0.260	0.238	0.353	1.67
10^6^	24.986	0.240	0.202	0.314	1.26
10^5^	26.500	0.544	0.145	0.563	2.12
10^4^	31.627	0.330	0.179	0.374	1.18
10^3^	32.733	0.678	0.322	0.751	2.29
10^2^	39.013	0.196	0.695	0.722	1.85
10^1^	39.800	0.218	0.128	0.253	0.64
10^0^	40.000	0.000	0.000	0.000	0.00

### Investigation of *Anaplasma capra* in field samples by LAMP, TaqMan qPCR, and nested PCR tests, and comparison of results

A total of 150 blood samples collected from cattle (n:25), sheep (n:25), goats (n:25), buffalo (n:25), dogs (n:25), and cats (n:25) were tested for *A. capra* using nested-PCR, LAMP, and TaqMan qPCR assays. *A. capra* was detected in two samples (1.33%, one cattle and one sheep) by nested PCR, four samples (2.66%, one cattle, two sheep, and one goat) by TaqMan qPCR, and five samples (3.33%, one cattle, two sheep, one goat, and one buffalo) by both colorimetric and conventional LAMP assays (Table 5). Two samples that were positive by *groEL*-nested PCR were also positive by TaqMan qPCR and LAMP. Additionally, all four samples that were identified as positive for TaqMan qPCR were also positive for LAMP. Detailed information on the molecular analysis of field samples is presented in Supplementary Table 1.

**Table 5:** Detection of *A. capra* in field samples (cattle, sheep, goat, buffalo, dog, and cats) based on *GroEL* by nested-PCR, TaqMan qPCR, colorimetric, and conventional LAMP

Methods	Number of tested samples	Number of positive samples	Number of negative samples
Nested-PCR	150	2	148
TaqMan qPCR	150	4	146
Conventional LAMP	150	5	145
Colorimetric LAMP	150	5	145

**Fig. 7:**
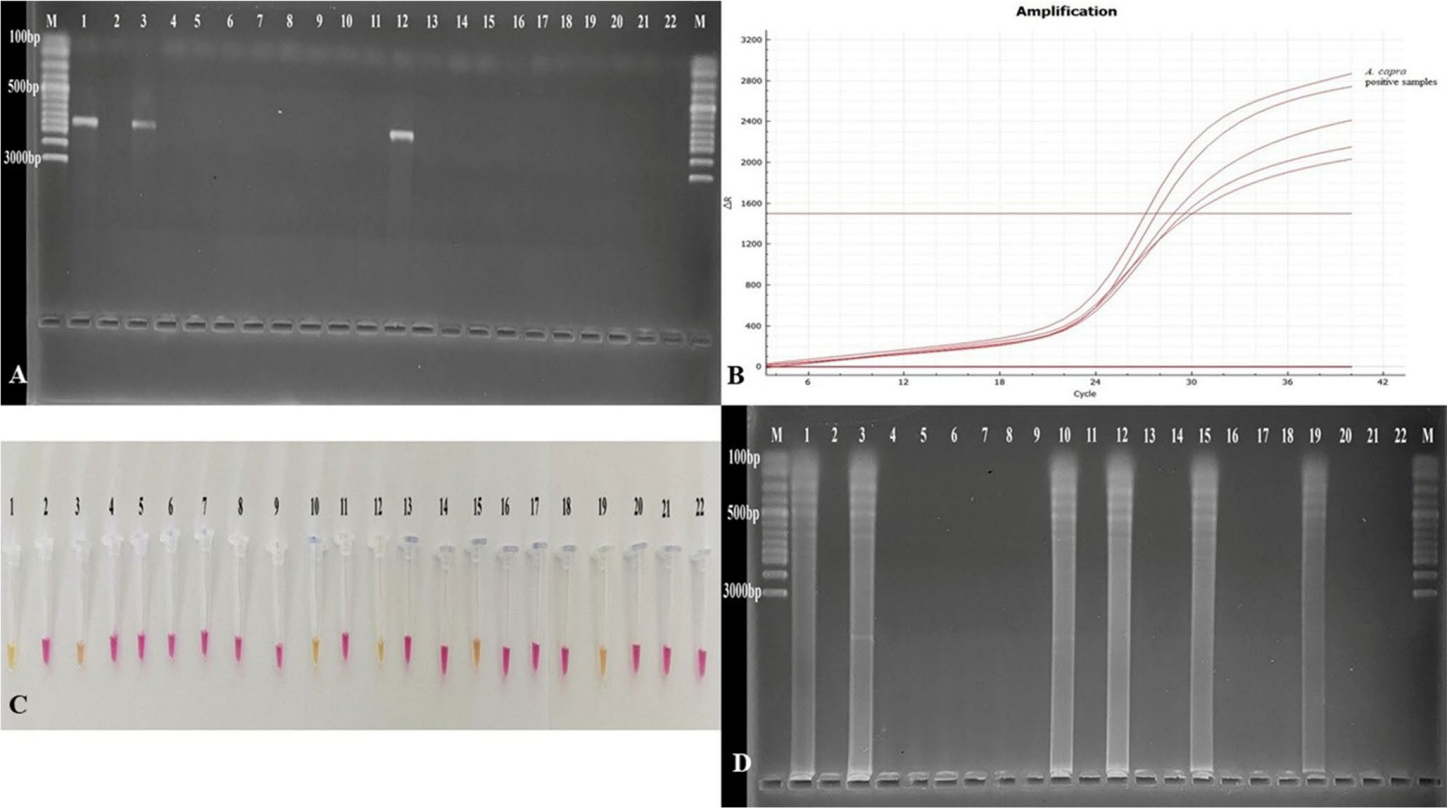
Investigation of *A. capra* in field samples. **A**: Nested-PCR results. M: marker, 1: positive control, 2: negative control, two positive samples [one cattle (3) and one sheep (12)]. **B**: TaqMan qPCR results. Positive samples [one positive control, four positive samples (one cattle, two sheep, and one goat)]. **C**: colorimetric LAMP and **D**: Conventional LAMP results. 1: positive control, 2: negative control, five positive samples [one cattle (3), two sheep (10 and 12), one goat (15), and one buffalo (19)]

The representative test results of 20 (including all the positive samples and four from each animal species) of the 150 studied are shown in Fig. 7. The results of samples 10, 15, and 19 were positive with both colorimetric and conventional LAMP, as illustrated in Fig. 7, but they were negative with *groEL*-nested PCR. The sample number 19 was positive in LAMP and negative in TaqMan qPCR. On the other hand, two samples, numbered 3 and 12, gave positive results in *groEL*-nested PCR as well as positive results with both LAMP and TaqMan qPCR. While the assay results are consistent with each other, LAMP appears to be more sensitive to the detection of *A. capra* in field samples than both *groEL*-nested PCR and TaqMan qPCR.

Cohen’s kappa test results revealed that the level of agreement values between nested-PCR and LAMP assay was κ = 0.58; the value was κ = 0.67 for nested-PCR and TaqMan qPCR assay, and κ = 0.89 for LAMP and TaqMan qPCR. These results demonstrate nearly perfect agreement between LAMP and TaqMan qPCR. Results regarding the evaluation of other results are presented in Table 6.

**Table 6:** Determination of the level of agreement between identification assays

Assays	Cohen’s kappa value	Level of agreement*
Nested-PCR vs. LAMP	0.58	Moderate
Nested-PCR vs. TaqMan qPCR	0.67	Substantial
LAMP vs. TaqMan qPCR	0.89	Almost perfect

*Levels of agreement values; 0.00–0.20.00.20; Slight, 0.21–0.40.21.40; Fair, 0.41–0.60.41.60; Moderate, 0.61–0.80.61.80; Substantial, 0.81–1.00.81.00; Almost perfect (Landis and Koch 1977)

## Discussion

Tick-borne pathogens can cause mild to severe infection in hosts, including domestic animals and humans, resulting in economic losses, reaching billions of dollars in the world each year (Lew-Tabor and Valle 2016). Species in the *Anaplasma* genus are important TBPs, and these pathogens may cause severe clinical symptoms and even death in various hosts (Kocan et al. 2010; Li et al. 2015; Sahin et al. 2022). *A. capra* is one of these species, and its detection generally relies on PCR-based molecular diagnostic methods (Li et al. 2015; Koh et al. 2018; Staji et al. 2021; Barradas et al. 2021; Elhachimi et al. 2021; Altay et al. 2022a; Remesar et al. 2022; Addo et al. 2023; Kumar et al. 2023; Lin et al. 2023; Sahin et al. 2023). Recent studies have shown that molecular-based methods have many advantages and disadvantages compared to each other in terms of specificity, sensitivity, limit of detection, cost, and duration (Notomi et al. 2000; Mori et al. 2001; Seo et al. 2018; Peng et al. 2021). In this study, LAMP (conventional and colorimetric) and TaqMan qPCR methods were developed and optimized for the diagnosis of *A. capra* in different hosts, and the results of these methods, such as sensitivity, specificity, LoD, and performance of these methods in field samples, were compared with the results of the *groEL*-nested PCR method.

Loop-Mediated Isothermal Amplification is a molecular diagnostic method that amplifies target DNA in the sample under isothermal conditions (Notomi et al. 2000). The most important advantages of the LAMP are that the method is completed in approximately one hour, it does not require expensive equipment, and its specificity and sensitivity are higher compared to other molecular methods (Notomi et al. 2000; Mori et al. 2001, 2009). Several LAMP assays have been developed to facilitate the diagnosis of vector-borne pathogens (Ikadai et al. 2004; Alhassan et al. 2007; Ma et al. 2011; Yang et al. 2016b; Wang et al. 2018; Giglioti et al. 2019). On the other hand, there is no LAMP method for the diagnosis of *A. capra*. As a result of optimization processes of the LAMP assay designed in this study, it was observed that *A. capra* could be diagnosed in 60 min at 62.0 °C (Fig. 1), but false positives were observed at times over 90 min (data not shown). The advantages of the LAMP method, such as the ability to work in laboratories with limited infrastructure, obtaining results in a short time, specificity and sensitivity, etc., and the results obtained within the aim of the study showed that both conventional and colorometric LAMP methods can be used successfully in the diagnosis of *A. capra* in hosts.

Real-time PCR method, which is fast, reliable, and highly specific and sensitive, has been successfully used in the diagnosis of several *Anaplasma* species (Reinbold et al. 2010; Dahmani et al. 2015; Díaz-Sánchez et al. 2020; Nkosi et al. 2025). Real-time PCR has been employed in only one investigation for the detection of *A. capra* (Song et al. 2020). Nevertheless, the genetic diversity of *A. capra* was only characterized in 2022. Consequently, the primers and probes used in the 2020 study do not cover all currently recognized genotypes, potentially resulting in false-negative detections (data not shown). In this study, TaqMan qPCR primer and probes were designed for the detection of *A. capra* genotypes in hosts (Table 2). The results obtained during the optimization process of the TaqMan qPCR assay (Figs. 2, 3 and 4D) have demonstrated that this method can be successfully used in the diagnosis of *A. capra*. The TaqMan qPCR efficiency value was calculated as 101.42%, and this value was found to be within acceptable ranges (90–110%) for qPCR (Rogers-Broadway and Karteris 2015). In this work, repeatability (intra-assay) and reproducibility (inter-assay) variation values were revealed, which a strong correlation between the tests (Table 4). This study also demonstrated that *A. capra* can be diagnosed much more quickly and reliably using a TaqMan qPCR assay (approximately one hour) compared to conventional PCR (at least two hours, maybe more). It is considered that this will also make an important contribution to researchers.

It is widely recognized that molecular-based approaches exhibit greater specificity and sensitivity than serological and microscopic procedures. Therefore, researchers have preferred molecular methods for the identification of pathogens, like *Anaplasma*, in hosts (Barradas et al. 2021; Staji et al. 2021; Altay et al. 2022a, b, c). Nevertheless, research has demonstrated that the specificity and sensitivity of molecular techniques may vary (Mori et al. 2009; Ma et al. 2011; Noden et al. 2018; Díaz-Sánchez et al. 2020; Foo et al. 2020). Specificity testing of the LAMP (both conventional and colorimetric) and TaqMan qPCR methods developed in this study was performed by both in-silico analysis (using the BLASTn database) and laboratory testing of common TBPs listed in Table 3. The specificity test findings of both methods indicated that they exclusively amplified *A. capra* and did not give cross-reactions with other pathogens (Fig. 4). In addition, the LoD of the TaqMan qPCR assay and LAMPs were investigated, and the results were compared to those of the PCR assay. The results obtained after these procedures showed that the LoD of the LAMP method was 10 times higher than qPCR and 100 times higher than conventional PCR (Fig. 5). Similar results were seen in sensitivity and specificity analyses of LAMP and qPCR methods developed for the diagnosis of *A. bovis* (Wang et al. 2018), A. *marginale* (Giglioti et al. 2019; Díaz-Sánchez et al. 2020), *Ehrlichia ruminantium* (Nakao et al. 2010), *Entamoeba histolytica* (Foo et al. 2020), and *Toxoplasma gondii* (Lin et al. 2012). Vector-borne pathogens such as *A. capra* can cause long-lasting, persistent infections in hosts (Kocan et al. 2010; Brown and Barbet 2016; Jouglin et al. 2019). Given that the amount of pathogen in the bloodstream of such animals may be quite low, the diagnosis of persistently infected animals can be difficult. However, persistently infected animals can become a source of infection for vectors in the environment. Consequently, vectors can facilitate the transmission of these infections to various hosts. It is believed that methods with high sensitivity should be employed to prevent this situation and to protect both animals and humans in the environment (Kocan et al. 2010; Brown and Barbet 2016). LAMPs and TaqMan qPCR methods with high sensitivity were developed by targeting the *groEL* gene for the diagnosis of *A. capra*, and it is thought that these methods will be useful in studies aimed at determining the species identification and epidemiology.

The diagnostic performance of the LAMP and TaqMan qPCR assays developed in this study was also tested on field samples. In this work, 150 field samples were researched, and as can be seen in Table 5, more positive results were obtained with LAMP than with TaqMan qPCR and *groEL*-nested PCR (Fig. 7). Furthermore, when the levels of agreement between assays were calculated with Cohen’s kappa test, consistency between the methods was also observed (Table 6). Furthermore, the study employed TaqMan qPCR and LAMPs to analyze blood samples from cattle, sheep, goats, buffaloes, dogs, and cats. The results indicated that DNA of above-mentioned animals was not amplified. When evaluated together with the LoD, these results show the superiority of its sensitivity along with other usage advantages in epidemiological studies of blood parasites such as *A. capra*. In this case, it is important in studies to determine the epidemiology of a species with a wide host spectrum, such as *A. capra*.

## Conclusion

In this study, for the first time, *groEL*-conventional and colorimetric LAMP and TaqMan qPCR assays were developed and optimized for the identification of *A. capra* in hosts. Data obtained during the optimization of the LAMP and TaqMan qPCR assay showed that both methods have higher specificity and sensitivity compared to conventional PCR. It has also been determined that both methods provide results much faster than PCR. LAMP and TaqMan qPCR assays, whose effectiveness is also determined in field studies using samples from different hosts, can make a great contribution to the understanding of the epidemiology of *A. capra*. Therefore, it is thought that these methods should be used in large-scale epidemiological studies involving more host species (such as various tick species and wild animals).

## Supplementary Information

Below is the link to the electronic supplementary material.

Supplementary Material 1

## Data Availability

All data generated or analyzed during this study are included in this manuscript.

## References

[CR1] Addo SO, Baako BOA, Bentil RE, Addae CA, Behene E, Asoala V, Sallam M, Mate S, Dunford JC, Larbi JA, Baidoo PK, Wilson MD, Diclaro IIJW, Dadzie SK (2023) Molecular survey of *Anaplasma* and *Ehrlichia* species in livestock ticks from Kassena-Nankana, Ghana; with a first report of *Anaplasma capra* and *Ehrlichia minasensis*. Arch Microbiol 205:92. 10.1007/s00203-023-03430-1 36795247 10.1007/s00203-023-03430-1

[CR2] Alhassan A, Thekisoe OM, Yokoyama N, Inoue N, Motloang MY, Mbati PA, Yin H, Katayama Y, Anzai T, Sugimoto C, Igarashi I (2007) Development of loop-mediated isothermal amplification (LAMP) method for diagnosis of equine piroplasmosis. Vet Parasitol 143:155–160. 10.1016/j.vetpar.2006.08.014 16973284 10.1016/j.vetpar.2006.08.014

[CR3] Altay K, Atas AD, Ozkan E (2017) Sivas yöresinde koyun keçi ve kenelerde *Theileria* ve *Babesia* türlerinin moleküler yöntemlerle araştırılması. Manas J Agr Vet Life Sci 7:30–39

[CR4] Altay K, Atas AD, Ograk YZ, Ozkan E (2020) Survey of *Theileria, Babesia* and *Anaplasma* infections of cattle and ticks from Sivas region of Turkey. Erciyes Üniv Vet Fak Derg 17:32–38. 10.32707/ercivet.690618

[CR5] Altay K, Erol U, Sahin OF (2022a) The first molecular detection of *Anaplasma capra* in domestic ruminants in the central part of Turkey, with genetic diversity and genotyping of *Anaplasma capra*. Trop Anim Health Prod 54:1–8. 10.1007/s11250-022-03125-7 10.1007/s11250-022-03125-7 35257219

[CR6] Altay K, Erol U, Sahin OF, Aytmirzakizi A, Temizel EM, Aydin MF, Dumanli N, Aktas M (2022b) The detection and phylogenetic analysis of *Anaplasma phagocytophilum*-like 1, *A*. *ovis* and *A*. *capra* in sheep: *A*. *capra* divides into two genogroups. Vet Res Commun 46:1271–1279. 10.1007/s11259-022-09998-1 36167934 10.1007/s11259-022-09998-1

[CR7] Altay K, Erol U, Sahin OF, Aytmirzakızı A (2022c) First molecular detection of <Emphasis Type="Italic">Anaplasma</Emphasis> species in cattle from Kyrgyzstan; molecular identification of human pathogenic novel genotype <Emphasis Type="Italic">Anaplasma capra</Emphasis> and <Emphasis Type="Italic">Anaplasma phagocytophilum</Emphasis> related strain. Ticks Tick Borne Dis 13:101861. 10.1016/j.ttbdis.2021.101861 34773849 10.1016/j.ttbdis.2021.101861

[CR8] Altay K, Erol U, Sahin OF (2024a) *Anaplasma capra*: a new emerging tick-borne zoonotic pathogen. Vet Res Commun 48:1329–1340. 10.1007/s11259-024-10337-9 38424380 10.1007/s11259-024-10337-9 PMC11147849

[CR9] Altay K, Abdugani A, Sahin OF, Muratova R, Erol U, Attokurov K, Abdurasulov I, Sakar HF, Risvanli A (2024b) A comprehensive molecular survey of vector-borne blood parasites in cattle in Kyrgyzstan with a note of the first molecular detection of *Anaplasma bovis* and *Candidatus* Anaplasma camelii. Trop Anim Health Prod 56:266. 10.1007/s11250-024-04112-w 39305339 10.1007/s11250-024-04112-w

[CR10] Altay K, Erol U, Sahin OF, Ulucesme MC, Aytmirzakizi A, Aktas M (2024c) Survey of tick-borne pathogens in grazing horses in Kyrgyzstan: phylogenetic analysis, genetic diversity, and prevalence of *Theileria equi*. Front Vet Sci 11:1359974. 10.3389/fvets.2024.1359974 38746933 10.3389/fvets.2024.1359974 PMC11091870

[CR11] Aubry P, Geale DW (2011) A review of bovine anaplasmosis. Transbound Emerg Dis 58:1–30. 10.1111/j.1865-1682.2010.01173.x 21040509 10.1111/j.1865-1682.2010.01173.x

[CR12] Barradas PF, Mesquita JR, Ferreira P, Gartner F, Carvalho M, Inacio E, Chivinda E, Katimba A, Amorim I (2021) Molecular identification and characterization of *Rickettsia* spp. and other tick-borne pathogens in cattle and their ticks from Huambo, Angola. Ticks Tick-borne Dis 12:101583. 10.1016/j.ttbdis.2020.101583 33160189 10.1016/j.ttbdis.2020.101583

[CR13] Brown WC, Barbet AF (2016) Persistent infections and immunity in ruminants to arthropod-borne bacteria in the family anaplasmataceae. Annu Rev Anim Biosci 4:177–197. 10.1146/annurev-animal-022513-114206 26734888 10.1146/annurev-animal-022513-114206

[CR14] Bustin SA, Benes V, Garson JA, Hellemans J, Huggett J, Kubista M, Mueller R, Nolan T, Pfaffl MW, Shipley GL, Vandesompele J, Wittwer CT (2009) The MIQE guidelines: minimum information for publication of quantitative real-time PCR experiments. Clin Chem 55:611–622. 10.1373/clinchem.2008.112797 19246619 10.1373/clinchem.2008.112797

[CR15] Casati S, Sager H, Gern L, Piffaretti JC (2006) Presence of potentially pathogenic *Babesia* sp. for human in *Ixodes ricinus* in Switzerland. Ann Agric Environ Med 13:65–70 16841874

[CR16] Cohen J (1960) A coefficient of agreement for nominal scales. Educ Psychol Meas 20:37–46. 10.1177/001316446002000104

[CR17] Dahmani M, Davoust B, Benterki MS, Fenollar F, Raoult D, Mediannikov O (2015) Development of a new PCR-based assay to detect Anaplasmataceae and the first report of *Anaplasma phagocytophilum* and *Anaplasma platys* in cattle from Algeria. Comp Immunol Microbiol Infect Dis 39:39–45. 10.1016/j.cimid.2015.02.002 25748051 10.1016/j.cimid.2015.02.002

[CR18] Díaz-Sánchez AA, Meli ML, Álvarez DO, Fonseca-Rodríguez O, Cabezas-Cruz A, Hofmann-Lehmann R, Corona-González B (2020) Development and application of a multiplex TaqMan® real-time qPCR assay for the simultaneous detection of <Emphasis Type="Italic">Anaplasma marginale</Emphasis> and <Emphasis Type="Italic">Theileria annulata</Emphasis> and molecular characterization of <Emphasis Type="Italic">Anaplasma marginale</Emphasis> from cattle in Western Cuba. Ticks Tick Borne Dis 11:101356. 10.1016/j.ttbdis.2019.101356 31870635 10.1016/j.ttbdis.2019.101356

[CR19] Dumanli N, Altay K, Aydin MF (2012) Tick species of cattle, sheep, and goats in Turkey. Turk Klin J Vet Sci 3:67–72

[CR20] Dumler JS, Barbet AF, Bekker CP, Dasch GA, Palmer GH, Ray SC, Rikihisa Y, Rurangirwa FR (2001) Reorganization of genera in the families rickettsiaceae and anaplasmataceae in the order rickettsiales: unification of some species of *Ehrlichia* with *Anaplasma*, *Cowdria* with *Ehrlichia* and *Ehrlichia* with *Neorickettsia*, descriptions of six new species combinations and designation of *Ehrlichia equi* and ‘HGE agent’ as subjective synonyms of *Ehrlichia phagocytophila*. Int J Syst Evol Microbiol 51:2145–2165. 10.1099/00207713-51-6-2145 11760958 10.1099/00207713-51-6-2145

[CR21] Elhachimi L, Rogiers C, Casaert S, Fellahi S, Van Leeuwen T, Dermauw W, Valcarcel F, Olmeda AS, Daminet S, Khatat SEH, Sahibi H, Duchateau L (2021) Ticks and tick-borne pathogens abound in the cattle population of the Rabat-Sale Kenitra Region, Morocco. Pathogens 10:1594. 10.3390/pathogens10121594 34959550 10.3390/pathogens10121594 PMC8703448

[CR22] Erol U, Sahin OF, Altay K (2022) Molecular survey of <Emphasis Type="Italic">Anaplasma phagocytophilum</Emphasis> and related strains in sheep and goats from Sivas; with a high prevalence of <Emphasis Type="Italic">Anaplasma phagocytophilum</Emphasis>-like 1. Turkiye Parazitol Derg 46:293–300. 10.4274/tpd.galenos.2022.47965 36444404 10.4274/tpd.galenos.2022.47965

[CR23] Foo PC, Nurul Najian AB, Muhamad NA, Ahamad M, Mohamed M, Yean Yean C, Lim BH (2020) Loop-mediated isothermal amplification (LAMP) reaction as viable PCR substitute for diagnostic applications: a comparative analysis study of LAMP, conventional PCR, nested PCR (nPCR) and real-time PCR (qPCR) based on *Entamoeba histolytica* DNA derived from faecal sample. BMC Biotechnol 20:1–15. 10.1186/s12896-020-00629-8 32571286 10.1186/s12896-020-00629-8 PMC7310076

[CR24] Giglioti R, Bassetto CC, Okino CH, de Oliveira HN, de Sena Oliveira MC (2019) Development of a loop-mediated isothermal amplification (LAMP) assay for the detection of *Anaplasma marginale*. Exp Appl Acarol 77:65–72. 10.1007/s10493-018-0327-y 30478537 10.1007/s10493-018-0327-y

[CR25] Grandi G, Aspan A, Pihl J, Gustafsson K, Engstrom F, Jinnerot T, Soderlund R, Chirico J (2018) Detection of tick-borne pathogens in lambs undergoing prophylactic treatment against ticks on two Swedish farms. Front Vet Sci 5:72. 10.3389/fvets.2018.00072 29713635 10.3389/fvets.2018.00072 PMC5911771

[CR26] Guo WP, Huang B, Zhao Q, Xu G, Liu B, Wang YH, Zhou EM (2018) Human-pathogenic <Emphasis Type="Italic">Anaplasma</Emphasis> spp., and <Emphasis Type="Italic">Rickettsia</Emphasis> spp. in animals in Xi’an, China. PLoS Negl Trop Dis 12:e0006916. 10.1371/journal.pntd.0006916 30419024 10.1371/journal.pntd.0006916 PMC6258427

[CR27] Han R, Yang JF, Mukhtar MU, Chen Z, Niu QL, Lin YQ, Liu GY, Luo JX, Yin H, Liu ZJ (2019) Molecular detection of *Anaplasma* infections in ixodid ticks from the Qinghai-Tibet Plateau Infect. Dis Poverty 8:83–90. 10.1186/s40249-019-0522-z 10.1186/s40249-019-0522-z PMC6366118 30728069

[CR28] Ikadai H, Tanaka H, Shibahara N, Matsuu A, Uechi M, Itoh N, Oshiro S, Kudo N, Igarashi I, Oyamada T (2004) Molecular evidence of infections with *Babesia gibsoni* parasites in Japan and evaluation of the diagnostic potential of a loop-mediated isothermal amplification method. J Clin Microbiol 42:2465–2469. 10.1128/JCM.42.6.2465-2469.2004 15184421 10.1128/JCM.42.6.2465-2469.2004 PMC427837

[CR29] Ishaq M, Ijaz M, Lateef M, Ahmed A, Muzammil I, Javed MU, Raza A, Ghumman NZ (2022) Molecular characterization of *Anaplasma capra* infecting captive mouflon (*Ovis gmelini*) and domestic sheep (*Ovis aries*) of Pakistan. Small Ruminant Res 16:106837. 10.1016/j.smallrumres.2022.106837

[CR31] Jouglin M, Blanc B, de la Cotte N, Bastian S, Ortiz K, Malandrin L (2019) First detection and molecular identification of the zoonotic *Anaplasma capra* in deer in France. PLoS One 14:e0219184. 10.1371/journal.pone.0219184 31276519 10.1371/journal.pone.0219184 PMC6611577

[CR30] Jouglin M, Blanc B, Brunet A, Ortiz K, Malandrin L (2025) <Emphasis Type="Italic">Anaplasma capra</Emphasis> and <Emphasis Type="Italic">Haemaphysalis concinna</Emphasis>: investigating a potential vector relationship in a wildlife reserve. Ticks Tick Borne Dis. 10.1016/j.ttbdis.2025.102500 40483925 10.1016/j.ttbdis.2025.102500

[CR32] Kawahara M, Rikihisa Y, Lin Q, Isogai E, Tahara K, Itagaki A, Hiramitsu Y, Tajima T (2006) Novel genetic variants of *Anaplasma phagocytophilum, Anaplasma bovis, Anaplasma centrale*, and a novel *Ehrlichia* sp. in wild deer and ticks on two major islands in Japan. Appl Environ Microbiol 72:1102–1109. 10.1128/AEM.72.2.1102-1109.2006 16461655 10.1128/AEM.72.2.1102-1109.2006 PMC1392898

[CR36] Khumalo ZTH, Brayton KA, Collins NE, Chaisi ME, Oosthuizen MC (2018) Evidence confirming the phylogenetic position of *Anaplasma centrale* (ex theiler 1911) Ristic and Kreier 1984. Int J Syst Evol Microbiol 68:2682–2691. 10.1099/ijsem.0 29916800 10.1099/ijsem.0.002832

[CR33] Kocan KM, de la Fuente J, Blouin EF, Coetzee JF, Ewing SA (2010) The natural history of *Anaplasma marginale*. Vet Parasitol 167:95–107. 10.1016/j.vetpar.2009.09.012 19811876 10.1016/j.vetpar.2009.09.012

[CR34] Koh FX, Panchadcharam C, Sitam FT, Tay ST (2018) Molecular investigation of *Anaplasma* spp. in domestic and wildlife animals in Peninsular Malaysia. Veterinary Parasitology: Regional Studies and Reports 13:141–147. 10.1016/j.vprsr.2018.05.006 31014863 10.1016/j.vprsr.2018.05.006

[CR35] Kubista M, Andrade JM, Bengtsson M, Forootan A, Jonák J, Lind K, Sindelka R, Sjöback R, Sjögreen B, Strömbom L, Ståhlberg A, Zoric N (2006) The real-time polymerase chain reaction. Mol Aspects Med 27:95–125. 10.1016/j.mam.2005.12.007 16460794 10.1016/j.mam.2005.12.007

[CR37] Kumar K, Singh K, Verma AK, Maurya PS, Prajapati MR, Kumar A, Sarkar TK (2023) Phylogenetic analysis and molecular characterization of field isolates of *Anaplasma* spp. from cattle in India. Vet Arh 93:535–548. 10.24099/vet.arhiv.1659

[CR38] Landis JR, Koch GG (1977) The measurement of observer agreement for categorical data. Biometrics 33:159–174. 10.2307/2529310 843571

[CR39] Lew AE, Gale KR, Minchin CM, Shkap V, de Waal DT (2003) Phylogenetic analysis of the erythrocytic <Emphasis Type="Italic">Anaplasma</Emphasis> species based on 16S rDNA and <Emphasis Type="Italic">groEL</Emphasis> (HSP60) sequences of <Emphasis Type="Italic">A. marginale, A. centrale</Emphasis>, and <Emphasis Type="Italic">A. ovis</Emphasis> and the specific detection of <Emphasis Type="Italic">A. centrale</Emphasis> vaccine strain. Vet Microbiol 92:145–160. 10.1016/s0378-1135(02)00352-8 12488078 10.1016/s0378-1135(02)00352-8

[CR40] Lew-Tabor AE, Valle MR (2016) A review of reverse vaccinology approaches for the development of vaccines against ticks and tick borne diseases. Ticks Tick-borne Dis 7:573–585. 10.1016/j.ttbdis.2015.12.012 26723274 10.1016/j.ttbdis.2015.12.012

[CR41] Li H, Zheng YC, Ma L, Jia N, Jiang BG, Jiang RR, Huo QB, Wang YW, Liu HB, Chu YL, Song YD, Yao NN, Sun T, Zeng FY, Dumler JS, Jiang JF, Cao WC (2015) Human infection with a novel tick-borne *Anaplasma* species in china: a surveillance study. Lancet Infect Dis 15:663–670. 10.1016/S1473-3099(15)70051-4 25833289 10.1016/S1473-3099(15)70051-4

[CR42] Lin Z, Zhang Y, Zhang H, Zhou Y, Cao J, Zhou J (2012) Comparison of loop-mediated isothermal amplification (LAMP) and real-time PCR method targeting a 529-bp repeat element for diagnosis of toxoplasmosis. Vet Parasitol 185:296–300. 10.1016/j.vetpar.2011.10.016 22051073 10.1016/j.vetpar.2011.10.016

[CR43] Lin ZT, Ye RZ, Liu JY, Wang XY, Zhu WJ, Li YY, Cui XM, Cao WC (2023) Epidemiological and phylogenetic characteristics of emerging *Anaplasma capra*: a systematic review with modeling analysis. Infect Genet Evol 115:105510. 10.1016/j.meegid.2023.105510 37778674 10.1016/j.meegid.2023.105510

[CR44] Liu Z, Ma M, Wang Z, Wang J, Peng Y, Li Y, Guan G, Luo J, Yin H (2012) Molecular survey and genetic identification of *Anaplasma* species in goats from central and Southern China. Appl Environ Microbiol 78:464–470. 10.1128/AEM.06848-11 22057867 10.1128/AEM.06848-11 PMC3255723

[CR45] Ma M, Liu Z, Sun M, Yang J, Guan G, Li Y, Luo J, Yin H (2011) Development and evaluation of a loop-mediated isothermal amplification method for rapid detection of *Anaplasma ovis*. J Clin Microbiol 49:2143–2146. 10.1128/JCM.02536-10 21471346 10.1128/JCM.02536-10 PMC3122716

[CR47] Mori Y, Notomi T (2009) Loop-mediated isothermal amplification (LAMP): a rapid, accurate, and cost-effective diagnostic method for infectious diseases. J Infect Chemother 15:62–69. 10.1007/s10156-009-0669-9 19396514 10.1007/s10156-009-0669-9 PMC7087713

[CR46] Mori Y, Nagamine K, Tomita N, Notomi T (2001) Detection of loop-mediated isothermal amplification reaction by turbidity derived from magnesium pyrophosphate formation. Biochem Biophys Res Commun 289:150–154. 10.1006/bbrc.2001.5921 11708792 10.1006/bbrc.2001.5921

[CR48] Nakao R, Stromdahl EY, Magona JW, Faburay B, Namangala B, Malele I, Inoue N, Geysen D, Kajino K, Jongejan F, Sugimoto C (2010) Development of loop-mediated isothermal amplification (LAMP) assays for rapid detection of *Ehrlichia ruminantium*. BMC Microbiol 10:1–1. 10.1186/1471-2180-10-296 21087521 10.1186/1471-2180-10-296 PMC3000401

[CR49] Nkosi NF, Byaruhanga C, Arega SM, Conan A, Knobel DL, Oosthuizen MC, Quan M (2025) Development and validation of a real-time PCR assay, and phylogenetic analysis of *Anaplasma platys*. Vet Parasitol 337:110475. 10.1016/j.vetpar.2025.110475 40286478 10.1016/j.vetpar.2025.110475

[CR50] Noden BH, Martin J, Carrillo Y, Talley JL, Ochoa-Corona FM (2018) Development of a loop-mediated isothermal amplification (LAMP) assay for rapid screening of ticks and fleas for spotted fever group rickettsia. PLoS One 13:e0192331. 10.1371/journal.pone.0192331 29390021 10.1371/journal.pone.0192331 PMC5794167

[CR51] Notomi T, Okayama H, Masubuchi H, Yonekawa T, Watanabe K, Amino N, Hase T (2000) Loop-mediated isothermal amplification of DNA. Nucleic Acids Res 28:e63-e63. 10.1093/nar/28.12.e63 10871386 10.1093/nar/28.12.e63 PMC102748

[CR52] Numan M, Alouffi A, Almutairi MM, Tanaka T, Ahmed H, Akbar H, Rashid MI, Tsai KH, Ali A (2023) First detection of *Theileria sinensis*-like and *Anaplasma capra* in *Ixodes kashmiricus*: with notes on *cox1*-based phylogenetic position and new locality records. Animals 13:3232. 10.3390/ani13203232 37893956 10.3390/ani13203232 PMC10603726

[CR53] Peng Y, Lu C, Yan Y, Shi K, Chen Q, Zhao C, Wang R, Zhang L, Jian F, Ning C (2021) The first detection of *Anaplasma capra*, an emerging zoonotic *Anaplasma* sp., in erythrocytes. Emerg Microbes Infect 10:226–234. 10.1080/22221751.2021.1876532 33446064 10.1080/22221751.2021.1876532 PMC7894429

[CR54] Reinbold JB, Coetzee JF, Sirigireddy KR, Ganta RR (2010) Detection of *Anaplasma marginale* and *A. phagocytophilum* in bovine peripheral blood samples by duplex real-time reverse transcriptase PCR assay. J Clin Microbiol 48:2424–2432. 10.1128/jcm.02405-09 20463162 10.1128/JCM.02405-09 PMC2897487

[CR55] Remesar S, Prieto A, Garcia-Dios D, Lopez-Lorenzo G, Martinez-Calabuig N, Diaz-Cao JM, Panadero R, Lopez CM, Fernandez G, Díez-Banos P, Morrondo P, Díaz P (2022) Diversity of *Anaplasma* species and importance of mixed infections in roe deer from Spain. Transbound Emerg Dis 69:e374–e385. 10.1111/tbed.14319 34529897 10.1111/tbed.14319

[CR56] Rogers-Broadway KR, Karteris E (2015) Amplification efficiency and thermal stability of qPCR instrumentation: current landscape and future perspectives. Exp Ther Med 10:1261–1264. 10.3892/etm.2015.2712 26622475 10.3892/etm.2015.2712 PMC4578049

[CR57] Sahin OF, Erol U, Altay K (2022) Buffaloes as new hosts for *Anaplasma capra*: molecular prevalence and phylogeny based on *gtlA, groEL*, and 16S rRNA genes. Res Vet Sci 152:458–464. 10.1016/j.rvsc.2022.09.008 36148715 10.1016/j.rvsc.2022.09.008

[CR58] Sahin OF, Erol U, Duzlu O, Altay K (2023) Molecular survey of *Anaplasma phagocytophilum* and related variants in water buffaloes: the first detection of *Anaplasma phagocytophilum*-like 1. Comp Immunol Microbiol Infect Dis 98:102004. 10.1016/j.cimid.2023.102004 37356166 10.1016/j.cimid.2023.102004

[CR59] Saratsis A, Ligda P, Aal F, Jelicic M, Polgar J, de Vries M, Mastranestasis I, Musella V, Rinaldi L, Jongejan F, Sotiraki S (2022) The scenario of ticks and tick-borne pathogens of sheep on a Mediterranean Island. Microorganisms 19:1551. 10.3390/microorganisms10081551 10.3390/microorganisms10081551 PMC9412349 36013969

[CR60] Scoles GA, Broce AB, Lysyk TJ, Palmer GH (2005a) Relative efficiency of biological transmission of *Anaplasma marginale* (Rickettsiales: Anaplasmataceae) by *Dermacentor andersoni* (Acari: Ixodidae) compared with mechanical transmission by *Stomoxys calcitrans* (Diptera: Muscidae). J Med Entomol 42:668–675. 10.1093/jmedent/42.4.668 16119558 10.1603/0022-2585(2005)042[0668:REOBTO]2.0.CO;2

[CR61] Scoles GA, Ueti MW, Palmer GH (2005b) Variation among geographically separated populations of *Dermacentor andersoni* (Acari: Ixodidae) in midgut susceptibility to *Anaplasma marginale* (Rickettsiales: Anaplasmataceae). J Med Entomol 42:153–162. 10.1093/jmedent/42.2.153 15799524 10.1603/0022-2585(2005)042[0153:vagspo]2.0.co;2

[CR62] Seo MG, Ouh IO, Lee H, Geraldino PJL, Rhee MH, Kwon OD, Kwak D (2018) Differential identification of *Anaplasma* in cattle and potential of cattle to serve as reservoirs of *Anaplasma capra*, an emerging tick-borne zoonotic pathogen. Vet Microbiol 226:15–22. 10.1016/j.vetmic.2018.10.008 30389039 10.1016/j.vetmic.2018.10.008

[CR63] Shi K, Li J, Yan Y, Chen Q, Wang K, Zhou Y, Li D, Chen Y, Yu F, Peng Y, Zhang Y, Ning C (2019) Dogs as new hosts for the emerging zoonotic pathogen *Anaplasma capra* in China. Front Cell Infect Microbiol 9:394. 10.3389/fcimb.2019.00394 31850236 10.3389/fcimb.2019.00394 PMC6901931

[CR64] Shi Y, Yang J, Guan G, Liu Z, Luo J, Song M (2020) Molecular investigation of *Anaplasma* species in sheep from Heilongjiang Province, northeast China identified four *Anaplasma* species and a novel genotype of *Anaplasma capra*. Parasitol Int 76:102072. 10.1016/j.parint.2020.102072 32044431 10.1016/j.parint.2020.102072

[CR65] Song J, Zhao S, Li Y, Wang H, Zhang L, Wang J, Ning C, Peng Y (2020) Duplex TaqMan real-time PCR assay for simultaneous detection and quantification of *Anaplasma capra* and *Anaplasma phagocytophilum* infection. Mol Cell Probes 49:101487. 10.1016/j.mcp.2019.101487 31731011 10.1016/j.mcp.2019.101487

[CR66] Staji H, Yousefi M, Hamedani MA, Tamai IA, Khaligh SG (2021) Genetic characterization and phylogenetic of *Anaplasma capra* in Persian onagers (*Equus hemionus onager*). Vet Microbiol 261:109199. 10.1016/j.vetmic.2021.109199 34385006 10.1016/j.vetmic.2021.109199

[CR67] Tamura K, Stecher G, Kumar S (2021) MEGA11: molecular evolutionary genetics analysis version 11. Mol Biol Evol 38:3022–3027. 10.1093/molbev/msab120 33892491 10.1093/molbev/msab120 PMC8233496

[CR68] Wang J, Zhang Y, Cui Y, Yan Y, Wang X, Wang R, Jian F, Zhang L, Ning C (2018) A rapid, simple and sensitive loop-mediated isothermal amplification method to detect *Anaplasma bovis* in sheep and goats samples. Parasitol Int 67:70–73. 10.1016/j.parint.2017.03.005 28351721 10.1016/j.parint.2017.03.005

[CR69] Yan Y, Wang K, Cui Y, Zhou Y, Zhao S, Zhang Y, Jian F, Wang R, Zhang L, Ning C (2021) Molecular detection and phylogenetic analyses of <Emphasis Type="Italic">Anaplasma</Emphasis> spp. in <Emphasis Type="Italic">Haemaphysalis longicornis</Emphasis> from goats in four provinces of China. Sci Rep 11:14155. 10.1038/s41598-021-93629-3 34238975 10.1038/s41598-021-93629-3 PMC8266805

[CR70] Yang J, Liu Z, Niu Q, Liu J, Han R, Liu G, Shi Y, Yin H (2016a) Molecular survey and characterization of a novel *Anaplasma* species closely related to *Anaplasma capra* in ticks, northwestern China. Parasit Vectors 9:1–5. 10.1186/s13071-016-1886-6 27884197 10.1186/s13071-016-1886-6 PMC5123347

[CR71] Yang Y, Li Q, Wang S, Chen X, Du A (2016b) Rapid and sensitive detection of *Babesia bovis* and *Babesia bigemina* by loop-mediated isothermal amplification combined with a lateral flow dipstick. Vet Parasitol 219:71–76. 10.1016/j.vetpar.2016.02.004 26921043 10.1016/j.vetpar.2016.02.004

[CR72] Zobba R, Anfossi AG, Pinna Parpaglia ML, Dore GM, Chessa B, Spezzigu A, Rocca S, Visco S, Pittau M, Alberti A (2014) Molecular investigation and phylogeny of *Anaplasma* spp. in mediterranean ruminants reveal the presence of neutrophil-tropic strains closely related to *A. platys*. Appl Environ Microbiol 80:271–280. 10.1128/AEM.03129-13 24162569 10.1128/AEM.03129-13 PMC3911010

