# Supplementary material for: Application of LAMP and TaqMan qPCR for the rapid diagnosis of Anaplasma Capra (an emerging tick-borne zoonotic pathogen) and comparison with Nested-PCR
Source: Vet Res Commun. 2026 Feb 9;50(2):153. doi: 10.1007/s11259-026-11074-x (PMC12886309; doi:10.1007/s11259-026-11074-x)
Supplement: Supplementary file 1 — Supplementary Material 1 [file 11259_2026_11074_MOESM1_ESM.docx]

| **Supplementary Table 1** Results from field samples examined using molecular methods in this study | | | | | |
| --- | --- | --- | --- | --- | --- |
| **Animals** | **Origin of Animals** | **Methods used in this study** | | | |
|  |  | **Nested-PCR** | **TaqMan qPCR** | **Colorimetric LAMP** | **Conventional LAMP** |
| Cattle-1 | Giresun | - | - | - | - |
| Cattle-2 | Giresun | - | - | - | - |
| Cattle-3 | Giresun | - | - | - | - |
| Cattle-4 | Giresun | - | - | - | - |
| Cattle-5 | Giresun | - | - | - | - |
| **Cattle-6** | **Giresun** | **+** | **+** | **+** | **+** |
| Cattle-7 | Giresun | - | - | - | - |
| Cattle-8 | Giresun | - | - | - | - |
| Cattle-9 | Giresun | - | - | - | - |
| Cattle-10 | Giresun | - | - | - | - |
| Cattle-11 | Giresun | - | - | - | - |
| Cattle-12 | Giresun | - | - | - | - |
| Cattle-13 | Giresun | - | - | - | - |
| Cattle-14 | Giresun | - | - | - | - |
| Cattle-15 | Giresun | - | - | - | - |
| Cattle-16 | Giresun | - | - | - | - |
| Cattle-17 | Giresun | - | - | - | - |
| Cattle-18 | Giresun | - | - | - | - |
| Cattle-19 | Giresun | - | - | - | - |
| Cattle-20 | Giresun | - | - | - | - |
| Cattle-21 | Giresun | - | - | - | - |
| Cattle-22 | Giresun | - | - | - | - |
| Cattle-23 | Giresun | - | - | - | - |
| Cattle-24 | Giresun | - | - | - | - |
| Cattle-25 | Giresun | - | - | - | - |
| Sheep-1 | Samsun | - | - | - | - |
| Sheep-2 | Samsun | - | - | - | - |
| Sheep-3 | Samsun | - | - | - | - |
| Sheep-4 | Samsun | - | - | - | - |
| Sheep-5 | Samsun | - | - | - | - |
| Sheep-6 | Samsun | - | - | - | - |
| Sheep-7 | Samsun | - | - | - | - |
| Sheep-8 | Samsun | - | - | - | - |
| Sheep-9 | Samsun | - | - | - | - |
| **Sheep-10** | **Samsun** | **+** | **+** | **+** | **+** |
| Sheep-11 | Samsun | - | - | - | - |
| Sheep-12 | Samsun | - | - | - | - |
| Sheep-13 | Samsun | - | - | - | - |
| Sheep-14 | Samsun | - | - | - | - |
| Sheep-15 | Samsun | - | - | - | - |
| Sheep-16 | Samsun | - | - | - | - |
| Sheep-17 | Samsun | - | - | - | - |
| Sheep-18 | Samsun | - | - | - | - |
| Sheep-19 | Samsun | - | - | - | - |
| Sheep-20 | Samsun | - | - | - | - |
| **Sheep-21** | **Samsun** | - | **+** | **+** | **+** |
| Sheep-22 | Samsun | - | - | - | - |
| Sheep-23 | Samsun | - | - | - | - |
| Sheep-24 | Samsun | - | - | - | - |
| Sheep-25 | Samsun | - | - | - | - |
| Goat-1 | Samsun | - | - | - | - |
| Goat-2 | Samsun | - | - | - | - |
| Goat-3 | Samsun | - | - | - | - |
| Goat-4 | Samsun | - | - | - | - |
| Goat-5 | Samsun | - | - | - | - |
| **Goat-6** | **Samsun** | **-** | **+** | **+** | **+** |
| Goat-7 | Samsun | - | - | - | - |
| Goat-8 | Samsun | - | - | - | - |
| Goat-9 | Samsun | - | - | - | - |
| Goat-10 | Samsun | - | - | - | - |
| Goat-11 | Samsun | - | - | - | - |
| Goat-12 | Samsun | - | - | - | - |
| Goat-13 | Samsun | - | - | - | - |
| Goat-14 | Samsun | - | - | - | - |
| Goat-15 | Samsun | - | - | - | - |
| Goat-16 | Samsun | - | - | - | - |
| Goat-17 | Samsun | - | - | - | - |
| Goat-18 | Samsun | - | - | - | - |
| Goat-19 | Samsun | - | - | - | - |
| Goat-20 | Samsun | - | - | - | - |
| Goat-21 | Samsun | - | - | - | - |
| Goat-22 | Samsun | - | - | - | - |
| Goat-23 | Samsun | - | - | - | - |
| Goat-24 | Samsun | - | - | - | - |
| Goat-25 | Samsun | - | - | - | - |
| Buffalo-1 | Sivas | - | - | - | - |
| Buffalo-2 | Sivas | - | - | - | - |
| Buffalo-3 | Sivas | - | - | - | - |
| Buffalo-4 | Sivas | - | - | - | - |
| Buffalo-5 | Sivas | - | - | - | - |
| Buffalo-6 | Sivas | - | - | - | - |
| Buffalo-7 | Sivas | - | - | - | - |
| Buffalo-8 | Sivas | - | - | - | - |
| Buffalo-9 | Sivas | - | - | - | - |
| Buffalo-10 | Sivas | - | - | - | - |
| Buffalo-11 | Sivas | - | - | - | - |
| Buffalo-12 | Sivas | - | - | - | - |
| Buffalo-13 | Sivas | - | - | - | - |
| Buffalo-14 | Sivas | - | - | - | - |
| Buffalo-15 | Sivas | - | - | - | - |
| **Buffalo-16** | **Sivas** | **-** | **-** | **+** | **+** |
| Buffalo-17 | Sivas | - | - | - | - |
| Buffalo-18 | Sivas | - | - | - | - |
| Buffalo-19 | Sivas | - | - | - | - |
| Buffalo-20 | Sivas | - | - | - | - |
| Buffalo-21 | Sivas | - | - | - | - |
| Buffalo-22 | Sivas | - | - | - | - |
| Buffalo-23 | Sivas | - | - | - | - |
| Buffalo-24 | Sivas | - | - | - | - |
| Buffalo-25 | Sivas | - | - | - | - |
| Cat-1 | Denizli | - | - | - | - |
| Cat-2 | Denizli | - | - | - | - |
| Cat-3 | Denizli | - | - | - | - |
| Cat-4 | Denizli | - | - | - | - |
| Cat-5 | Denizli | - | - | - | - |
| Cat-6 | Denizli | - | - | - | - |
| Cat-7 | Denizli | - | - | - | - |
| Cat-8 | Denizli | - | - | - | - |
| Cat-9 | Denizli | - | - | - | - |
| Cat-10 | Denizli | - | - | - | - |
| Cat-11 | Denizli | - | - | - | - |
| Cat-12 | Denizli | - | - | - | - |
| Cat-13 | Denizli | - | - | - | - |
| Cat-14 | Denizli | - | - | - | - |
| Cat-15 | Denizli | - | - | - | - |
| Cat-16 | Denizli | - | - | - | - |
| Cat-17 | Denizli | - | - | - | - |
| Cat-18 | Denizli | - | - | - | - |
| Cat-19 | Denizli | - | - | - | - |
| Cat-20 | Denizli | - | - | - | - |
| Cat-21 | Denizli | - | - | - | - |
| Cat-22 | Denizli | - | - | - | - |
| Cat-23 | Denizli | - | - | - | - |
| Cat-24 | Denizli | - | - | - | - |
| Cat-25 | Denizli | - | - | - | - |
| Dog-1 | İstanbul | - | - | - | - |
| Dog-2 | İstanbul | - | - | - | - |
| Dog-3 | İstanbul | - | - | - | - |
| Dog-4 | İstanbul | - | - | - | - |
| Dog-5 | İstanbul | - | - | - | - |
| Dog-6 | İstanbul | - | - | - | - |
| Dog-7 | İstanbul | - | - | - | - |
| Dog-8 | İstanbul | - | - | - | - |
| Dog-9 | İstanbul | - | - | - | - |
| Dog-10 | İstanbul | - | - | - | - |
| Dog-11 | İstanbul | - | - | - | - |
| Dog-12 | İstanbul | - | - | - | - |
| Dog-13 | İstanbul | - | - | - | - |
| Dog-14 | İstanbul | - | - | - | - |
| Dog-15 | İstanbul | - | - | - | - |
| Dog-16 | İstanbul | - | - | - | - |
| Dog-17 | İstanbul | - | - | - | - |
| Dog-18 | İstanbul | - | - | - | - |
| Dog-19 | İstanbul | - | - | - | - |
| Dog-20 | İstanbul | - | - | - | - |
| Dog-21 | İstanbul | - | - | - | - |
| Dog-22 | İstanbul | - | - | - | - |
| Dog-23 | İstanbul | - | - | - | - |
| Dog-24 | İstanbul | - | - | - | - |
| Dog-25 | İstanbul | - | - | - | - |
| **Total** |  | **2 (1.33%)** | **4 (2.67%)** | **5 (3.33%)** | **5 (3.33%)** |
